# Macrophages of the Cardiorenal Axis and Myocardial Infarction

**DOI:** 10.3390/biomedicines11071843

**Published:** 2023-06-27

**Authors:** Maria Kercheva, Vyacheslav Ryabov, Aleksandra Gombozhapova, Ivan Stepanov, Julia Kzhyshkowska

**Affiliations:** 1 Cardiology Division, Siberian State Medical University, 2 Moscovsky Trakt, 634055 Tomsk, Russia; 2 Cardiology Research Institute, Tomsk National Research Medical Center of the RAS, 111a Kievskaya Street, 634012 Tomsk, Russia; 3 Laboratory of Translational and Cellular Biomedicine, National Research Tomsk State University, 36 Lenin Avenue, 634050 Tomsk, Russia; 4 Institute of Transfusion Medicine and Immunology, University of Heidelberg, 1-3 Theodor-Kutzer Ufer, 68167 Mannheim, Germany

**Keywords:** myocardial infarction, cardiac remodeling, cardiac macrophages, kidney macrophages, inflammation

## Abstract

The aim of our study was to compare the features of macrophage (mf) composition of the kidneys in patients with fatal myocardial infarction (MI) and in patients without cardiovascular diseases (CVD). We used kidney fragments taken during autopsy. Macrophage infiltration was assessed by immunohistochemistry: antibodies CD68 were used as a common mf marker, CD80—M1 type mf marker, CD163, CD206, and stabilin-1—M2 type. Macrophage composition of the kidneys in patients with fatal MI was characterized by the predominance of CD163+ cells among studied cells, and the control group was characterized by the predominance of CD163+, CD206+, and CD68+. In patients with MI, biphasic response from kidney cells was characterized for CD80+ and CD206+: their number decreased by the long-term period of MI; other cells did not show any dynamics. The exact number of CD80+ cells in kidneys of individuals without CVD was slightly higher than in patients with MI, and the number of CD206+—strikingly predominant. Subsequent analysis of CD80+ and CD206+ cells in a larger sample, as well as comparison of data with results obtained from survivors of MI, may bring us closer to understanding whether the influence on these cells can serve as a new target in personalized therapy in postinfarction complications.

## 1. Introduction

Impaired hemodynamics and inadequate renal perfusion cause the formation of cardiorenal syndrome (CRS) in myocardial infarction (MI) [1]. Acute impairment of kidney function in patients with MI develops in 7.1–29.3% of cases, worsens both short-term and long-term prognosis in this cohort of patients, and increases hospitalization time [2]. The existing understanding of CRS within its classification according to clinical and anamnestic data can detect cardiorenal dysfunction but does not change disease management and prognosis [3,4]. The cardiorenal relationships at the cellular level are poorly studied [5] and the existing experimental data are not sufficient to affect the course of the disease. Recently, it has become evident that cells of innate immunity are important to maintain a balanced relationship between the heart and the kidneys [5,6]. Changed polarization of macrophages (mf) in the kidneys induced by ischemia enhances the release of granulocyte–macrophage colony-stimulating factor into the bloodstream, which in turn causes subsequent polarization of myocardial mf into the regenerative M2 type and is associated with the development of fibrosis and adaptive myocardial hypertrophy [5]. However, clinical data on this interaction of innate immune cells along the heart–kidney axis are limited [7,8]. In our previous study, we investigated the composition of mf in the kidneys and its relationship with changes in mf infiltration into the heart, and with an adverse course of the disease in patients with fatal MI. A pronounced heterogeneity of the mf composition in the kidneys with the predominance of CD163+ cells was revealed in this cohort of patients. Among all studied cells we revealed two types—CD206+ and CD80+, which showed significant quantitative dynamics in the late period after MI and a number of relationships with both adverse prognosis and cardiac macrophages [9]. Yet, it remains unclear whether our data indicate changes in the mf composition of the kidneys in response to myocardial ischemia, and whether these changes can affect the course and prognosis of the disease. In this regard, the aim of the study was to compare data on the features of the mf composition in the kidneys in patients with fatal MI and in patients from the control group without a history of cardiovascular diseases (CVD).

## 2. Materials and Methods

### 2.1. Clinical and Anamnestic Characteristics

The study involved patients with fatal type 1 MI. The exclusion criteria were type II–V MI, oncological disorders, infectious complications (sepsis, pneumonia), and valvular defects requiring surgical intervention. The study protocol was approved by the Biomedical Ethics Committee of Cardiology Research Institute (Tomsk, Russia), protocol No. 128, of 23 December 2014. The study was conducted in accordance with the principles of the Declaration of Helsinki. Pathological autopsy was carried out in accordance with the order of the Ministry of Health of the Russian Federation of 6 June 2013, No. 354n, on the procedure of postmortem examinations. In this study, informed consent could not be obtained, yet this did not contradict the principles for conducting the study in accordance with the Declaration of Helsinki (informed consent, paragraph 32). 

The study object was kidney fragments taken from MI patients (n = 30) and from patients in the control group (n = 8) during autopsy. The control group consisted of people who died from fatal injuries and did not have CVD (aged from 18 to 55). An autopsy was performed within 24 h after death. The material was fixed in 10% buffered formalin for 1 day. The material for histological examination was prepared by standard method using a Thermo Scientific Excelsior AS (Thermo Fisher Scientific, Waltham, MA, USA). After that, the material was embedded in paraffin using a Tissue-Tek^®^ TEC™ 6 embedding console system (Sakura, Tokyo, Japan). The results were obtained using the equipment of the Center for Collective Use “Medical Genomics”, Tomsk National Medical Research Center.

To study the spatiotemporal pattern of accumulation of mf in the kidneys and their phenotypes, the patients with MI were divided into groups depending on infarction duration: group 1 involved those who died during the first three days after MI, within 72 h; group 2 included patients who died on day 4–28. 

We have already reported clinical and anamnestic data on these groups of patients [9]. It should be noted that the average age of the examined patients was 74.8 ± 9.8. The time from the onset of the disease to admission to the hospital was 180 min (120–720 min.). Circular MI was recorded in 40% of cases, and recurrent MI was observed in half of the patients. Heart failure (HF) in anamnesis occurred in 50% of patients. Cardiogenic shock was the main death factor; other reasons were cardiac rupture and arrhythmogenic shock.

### 2.2. Immunohistochemical Analysis 

To perform an immunohistochemical analysis with a rotary microtome (HM 355S, Thermo Fisher Scientific, Waltham, MA, USA), kidney sections were prepared: 10 sections from each block. The material was then applied to L-polylysine-coated slides, two sections per slide. The study was performed using an automated immunostainer (Leica Bond-Max, Wetzlar, Germany) in accordance with a standard protocol [10]. The study used antibodies against mf markers. CD68 was used as a common mf marker. CD80 was used as M1 type mf marker. CD163 and CD206 were used as classical M2 type mf markers, and stabilin-1 was used as an additional M2 type mf marker. We used mouse monoclonal antibodies against CD68 (Cell Marque, 1:500 dilution), antibodies against CD163 (Cell Marque, Rocklin, CA, USA, 1:50 dilution), antibodies against CD80 (Invitrogen, Waltham, MA, USA, 1:600 dilution), mouse monoclonal antibodies against CD206 (Santa Cruz, 1:100 dilution), and antibodies against stabilin-1 RS1 (1:1000 dilution) synthesized in the Laboratory of Innate Immunity and Immunological Tolerance (University of Heidelberg).

The studied markers were visualized using the BOND Polymer Refine Detection system (Leica, Wetzlar, Germany). BOND Polymer Refine Detection contains a peroxide block, post-primary, polymer reagent, DAB chromogen, and hematoxylin counterstain. Two independent experts counted positively stained mfs in the kidney and analyzed 10 randomly chosen fields of view (40× objective) using a Zeiss Axio Imager M2 microscope, bright field. 

### 2.3. Statistical Analysis

The obtained data were analyzed using the STATISTICA 12.0 software package. The quantitative data were tested for normality using the Shapiro–Wilk test. All quantitative indicators that showed abnormal distribution were described by the median (Me) and interquartile interval (Q1; Q3). The Mann–Whitney test was used to compare quantitative indicators in independent groups, Kendall rank correlation coefficient and Wilcoxon signed-rank test were used to dependent groups. Statistical hypotheses were tested with the significance value *p* = 0.05.

## 3. Results

The features of mf composition in the kidneys and its dynamics in MI patients were revealed through the analysis of the data obtained from both the control group and MI patients at different time periods—early and late periods after MI (Table 1).

**Figure 1:**
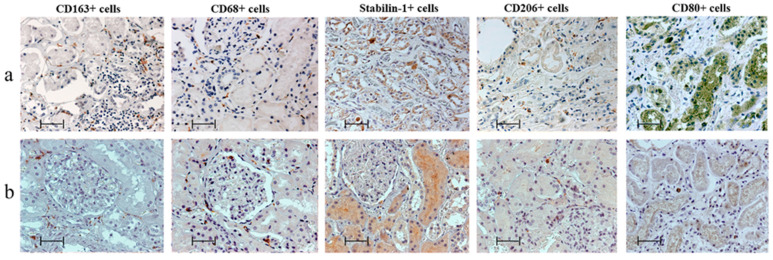
Features of macrophage infiltration of kidneys (**a**) in patients with myocardial infarction and (**b**) of control group, immunohistochemistry; scale bar: 50 µm.

**Figure 2:**
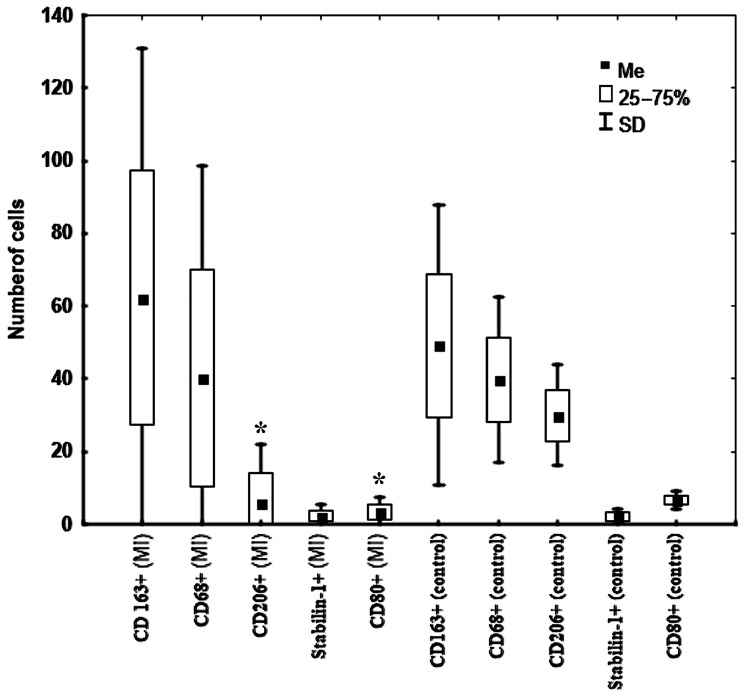
Features of the macrophage composition in the kidneys in patients with fatal MI (n = 30) and in patients from the control group (n = 8). Note: * —significant difference between patients with fatal MI from group 1 and group 2. Abbreviation: MI—myocardial infarction.

CD163+ cells were predominant in the kidneys of MI patients; however, in the control group these cells were predominant with CD68+ and CD206+ cells (Figure 1 and Figure 2).

Interestingly, among the studied kidney cells, CD206+ and CD80+ were the types of cells that had dynamics that changed depending on the MI period (Table 1). The number of these cells in the kidneys decreased in the late period after MI. The number of these cells in the kidneys of patients from the control group exceeded that in MI patients (Table 1, Figure 2). Other kidney cells neither changed over time nor differed from those in patients from the control group.

## 4. Discussion

Our data are unique and novel since we are the first to reveal the features of changes in the kidney mf composition in MI patients, and show the relationship between these changes and unfavorable outcome through comparison of these changes with the results obtained for patients without CVD. However, the results mainly indicate pathophysiological processes that occur under experimental ischemic conditions in animals.

Among all the studied cells, CD163+ predominate in terms of their number in the kidneys of patients with MI and they serve as one of the leading cell types in the control group. The CD163 cellular receptor is actively expressed on both monocytes and mf, and serves as a marker for alternatively activated M2 mf [11]. Monocytes are known to express a small amount of CD163. However, mf, particularly in the inflammation resolution phase, exhibit a high expression of CD163 [12]. As previously reported, a high concentration of these cell types in kidney biopsy specimens from patients with lupus nephritis [13] was due to the unfavorable course and prognosis of the disease. Similar data were reported for patients with IgA nephropathy [14] and for patients after kidney transplantation [15]. A high concentration of these cells in the myocardium in the late postinfarction period was also due to unfavorable outcome [16]. However, we assume that these cells are most likely involved in tissue homeostasis, immunological regulation, and tissue regeneration in case of any injury, and initiation, which explains their high and comparable concentration in kidney tissues in both groups [7]. 

The number of CD68+ cells is the next largest in the group of MI and they also serve as one of the leading cell types in the control group. CD68 is an immunohistochemical marker of the common population of mf, of which the main function is the absorption of apoptotic and damaged cells [17]. This can be the cause of a high concentration of these cells in MI patients. A high concentration of this cell type in kidneys in the late observation period probably shows their involvement in a prolonged inflammatory reaction and is associated with unfavorable prognosis, which indicates involvement of the innate immune system in postinfarction kidney regeneration. In a number of studies, concentration of CD68+ cells correlated with albuminuria and unfavorable outcome [18]. The number of these cells in MI patients from the studied group was comparable to the number of CD68+ cells in the kidneys of patients with a reduced glomerular filtration rate and the presence of lupus nephritis [19]. Yet, the number of cells was similar to that in healthy individuals, which may indirectly indicate the lack of the impact of this cell type on cardiorenal relationships in MI patients.

One of the most widely studied markers of M1 mf is CD80 [20]. M1 mf secrete pro-inflammatory cytokines, cathepsins, and matrix metalloproteinases, which induce the elimination of cellular debris and apoptotic cells and the beginning of the repair process [21]. The decrease in the level of these cells in patients with MI by the late period of MI determines the physiological course of the inflammatory response [21]. However, the fact that the level of CD80 cells is lower in individuals with MI than in the control group, along with a decrease in the level of M2 mf– CD206+ cells by the late period of MI and becoming comparable with CD80+ and stabilin-1 cells, may cause an adverse course of the disease. An inadequate weak inflammatory response, along with an inadequate low-intensity regeneration phase, can cause an unfavorable outcome of MI.

The study of the concentration of stabilin-1+ cells in the kidneys of patients with MI and without CVD showed single stabilin+ cells. Studies on the role of stabilin-1+ cells in cardiovascular pathology are insufficient [16,22]. Some data were obtained for patients with cancer [23]. The study of these cells in the kidneys of MI patients has not been performed. A low content of cells in the renal tissue of the examined MI patients probably indicates a negligible impact, or lack thereof, of these cells on the cardiorenal relationships. The data obtained in this study show inadequacy of the dichotomous model for assessing mf and their functions with regard to their conditional division into two phenotypes—pro- and anti-inflammatory. The behavior of all the studied M2 type mf is completely different. They are different in their concentration in tissues and in their response to myocardial ischemia. 

In our opinion, among all the studied kidney mf, CD206+ cells belonging to M2 type mf are most interesting and promising for further research [24]. This is the type of mf that showed significant dynamics in renal tissue in the late period after MI, as well as a significantly lower concentration in patients with fatal MI. A number of researchers reported data that are partly comparable to our data. In particular, comparison of the number of these cells in the kidneys of patients with acute interstitial nephritis and in a cohort of patients with acute tubular necrosis showed a lower number of CD206+ cells in patients with acute tubular necrosis [25]. This type of cells was referred to as a resident type of mf [25] involved in renal tissue homeostasis; therefore, their number can reduce under ischemic conditions and trigger an unfavorable course of the disease. In addition, according to the experimental data on rodents [5], the polarization of kidney mf under ischemic conditions, including CD206+ cells, stimulates the polarization of myocardial mf into M2 type, followed by the development of adaptive left ventricular hypertrophy and myocardial fibrosis. The effect of these processes on the disease outcome remained unclear. However, it should be noted that IL-4, IL-13, and IL-10 play a central role in the polarization of mfs in the direction of the M2 type [7]. We cannot currently confirm or deny these data, because the lack of a comprehensive, systematic analysis, including an analysis of the microenvironment, as well as the level of circulating markers, is a limitation of our study, the elimination of which is planned by us in the subsequent data collection. In addition to the influence of the microenvironment on the cell phenotype, a metabolic factor such as hypoxia may explain the low functional activity of CD206+ cells in kidneys tissue in patients with MI, which was different from that in patients without CVD [26]. It is interesting that the number of CD206+ cells in persons with MI was several times lower than in persons from the control group. An adequate/greater number of M2 mf in the kidneys may be required to change the polarization of cardiac mf in the early period after MI, which may affect the inflammatory response in the early postinfarction period and lead to unfavorable outcome. The number of these cells in MI patients was twice as low as that in patients with tubulointerstitial necrosis [25], which may indicate active involvement of this cell type in cardiorenal relationships under ischemic conditions. According to our previous data, the number of CD206+ cells in MI patients with CKD+ and CKD- was different, and the results of the multivariate analysis confirm our assumption about the relevance of studying this cell type as one of the key components of cardiorenal relationships under ischemic conditions [9]. The subsequent detailed analysis of the most pronounced pathological mf, and comparison of the data obtained on the autopsy material with those obtained for patients who have successfully come through MI, will help identify a specific target. This will contribute to targeted therapy and improve the prognosis for patients with MI affecting the development, course, and progression of postinfarction heart and renal failure.

### Limitation

This study was conducted as a single-center trial and the size of a sample was small. In addition, the ratio of kidney/myocardial mf at a given time period was not a static indicator. Nowadays, this work is of a fundamental and descriptive nature. Therefore, one promising direction for future research is the changes in the amount of cells with these phenotypes in vivo in patients with MI and favorable outcome. Future investigation needs to have a complex systematic approach, accessing the cells and microenvironments changes in the heart and comparing them with the changes in target organs, with a connection with circulated biomarkers and unfavorable outcomes of MI. For these reasons, further studies are required.

## 5. Conclusions

Macrophage composition of the kidneys in patients with fatal MI among all the cells studied by us was characterized by the predominance of CD163+ cells, the control group characterized by the predominance of CD163+, CD206+, and CD68+. In patients with fatal MI, biphasic response from kidney macrophages was characterized for CD80+ and CD206+ cells: their number decreased by the long-term period of MI; other cells did not show any dynamics. Moreover, the exact number of CD80+ cells in kidneys of individuals without cardiovascular disease was slightly higher than in fatal MI, and the number of CD206+ cells was strikingly predominant. Subsequent analysis of CD80+ and CD206+ cells in a larger sample, as well as comparison of data with results obtained from survivors of MI, may bring us closer to understanding whether the influence on these cells can serve as a new target in personalized therapy in postinfarction complications.

## Figures and Tables

**Table 1:** Features of macrophage composition in the kidneys in patients with fatal MI and in patients from the control group (number of positive stained macrophages counted per histology section area of interest and analyzed in 10 randomly chosen fields of view).

Cells	All Patients (n = 30)	Control (n = 8)	p1	Group 1 (n = 17)	Group 2 (n = 13)	p2	p3	p4
**CD163+**	55 (32; 97)	47 (34; 68)	0.5	55 (34; 72)	58 (32; 97)	0.5	0.9	0.4
**CD68+**	30 (23; 51) *	41 (33;48)	0.45	30 (24; 49) *	35(23; 51) *	0.7	0.4	0.7
**CD206+**	4 (2; 6) *, **	30 (27; 35)	0.0005	6 (5; 8)*,**	2 (1; 2) *, **	0.01	0.005	0.0001
**Stabilin-1+**	2 (1; 3) *, **, ***	2 (1; 3) *, **, ***	0.8	1 (1; 4) *, **, ***	2 (1; 2) *, **	0.3	0.9	0.7
**CD80+**	3 (2; 5) *, **, ***	6 (6; 7) *, **, ***, ****	0.0003	5 (3; 5) *, **, ***	2 (1;2) *, **	0.01	0.0005	0.0007

Note: MI—myocardial infarction. p1—Significant difference between the group with fatal MI and the control group; p2—significant difference between patients with fatal MI from group 1 and group 2; p3—significant difference between patients with control group from group 1; p4—significant difference between patients with control group from group 2. *—Significant difference in the group between the number of CD163+ cells and other cells; **—significant difference in the group between the number of CD68+ cells and other cells; ***—significant difference in the group between the number of CD206+ cells and other cells; ****—significant difference in the group between the number of stabilin-1+ cells and other cells.

## Data Availability

The data presented in this study are available upon request from the corresponding author.
